# American Cutaneous Leishmaniasis in Panama: a historical review of entomological studies on anthropophilic *Lutzomyia* sand fly species

**DOI:** 10.1186/1756-3305-7-218

**Published:** 2014-05-11

**Authors:** Larissa C Dutari, Jose R Loaiza

**Affiliations:** 1 Centro de Biodiversidad & Descubrimiento de Drogas, Instituto de Investigaciones Científicas & Servicios de Alta Tecnología, Edificio 219, Clayton, PO 0843–01103, Ciudad del Saber, República de Panamá; 2 Department of Biotechnology, Acharya Nagarjuna University, Guntur, India; 3 Programa Centroamericano de Maestría en Entomología, Vicerrectoría de Investigación & Postgrado, Universidad de Panamá, Ciudad de Panamá, República de Panamá

**Keywords:** *Leishmania*, *Lutzomyia*, Anthropophilic, Taxonomy, Bionomics, Vector control, Panama

## Abstract

We review existing information on the epidemiology of American Cutaneous Leishmaniasis (ACL) in Panama, with emphasis on the bionomics of anthropophilic *Lutzomyia* sand fly species. Evidence from Panamanian studies suggests that there are six anthropophilic species in the country: *Lutzomyia trapidoi*, *Lu. panamensis*, *Lu. gomezi*, *Lu. ylephiletor*, *Lu. sanguinaria* and *Lu. pessoana* (Henceforth *Lu. carrerai thula*). In general, these taxa are abundant, widespread and feed opportunistically on their hosts, which make them potential transmitters of pathogens to a broad range of wildlife, domesticated animals and humans. Furthermore, nearly all man-biting species in Panama (with the exception of *Lu. gomezi)* expand demographically during the rainy season when transmission is likely higher due to elevated *Leishmania* infection rates in vector populations. Despite this, data on the distribution and prevalence of ACL suggest little influence of vector density on transmission intensity. Apart from *Lu. trapidoi*, anthropophilic species seem to be most active in the understory, but vertical stratification, as well as their opportunistic feeding behavior, could vary geographically. This in turn seems related to variation in host species composition and relative abundance across sites that have experienced different degrees of human alteration (e.g., deforestation) in leishmaniasis endemic regions of Panama.

## Background

**Figure 1:**
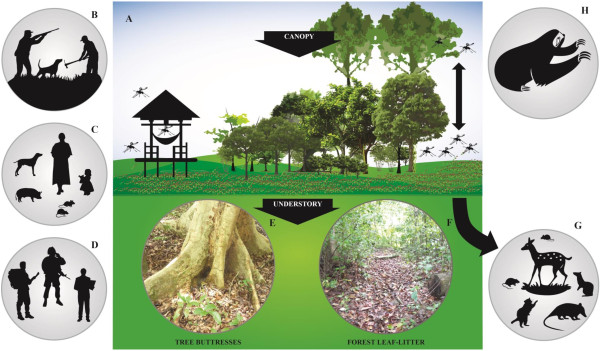
**Epidemiological patterns of *Leishmania *** **(*Viannia*) *panamensis *** **in Panama: (A) Sylvatic focus of American Cutaneous Leishmaniasis; (B) Non-stable transmission – farmers and hunters; (C) Endemic transmission – indigenous communities; (D) Epidemic transmission – military personnel, tourists and teachers; (E) Tree buttresses; (F) Forest leaf-litter; (G) Potential secondary reservoirs *Proechimys semispinosus *** **(Spiny rat), *Didelphis marsupialis *** **(Opossum), *Dasyprocta punctata *** **(Agoutis), *Odocoileus virginianus *** **(White-tail deer); and (H) Primary reservoir *Choloepus hoffmanni *** **(Two-toed sloth).**

American Cutaneous Leishmaniasis (ACL) is a Neotropical infection caused by unicellular parasites in the genus *Leishmania* (Kinetoplastida: Trypanosomatidae) and transmitted by insect vectors in the genus *Lutzomyia* (Psychodidae: Phlebotominae) [1-3]. This disease persists endemically in forested areas of Panama where it represents a major health problem for children, who suffer severe skin lesions and face disfigurement following infection [4]. In addition, customs such as hunting and farming put middle-aged men at greater risk of transmission in non-endemic unstable settlements [5,6]. Epidemic cycles begin when a group of immunologically naïve people such as military personnel, school teachers, scientists or international tourists enter sylvatic foci [6-15] [Figure 1].

**Figure 2:**
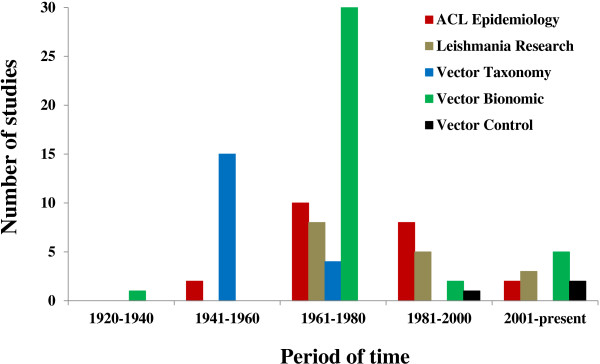
**The chart shows the number of studies carried out in Panama about American Cutaneous Leishmaniasis (ACL) and the bionomic (ecology + behavior) of anthropophilic *Lutzomyia *sand fly species from 1920 to the present.** The information is broken down by time period and research area (in numbers at the bottom and words and different colors on the right side, respectively).

Entomological research on ACL in Panama dates to the beginning of the 20^th^ century when the first occurrence records of sand flies (then known as *Phlebotomus*) were made in the country [16]. Graham Bell Fairchild and Marshall Hertig studied the natural history and systematics of sand fly species, describing several new taxa from various locales across Panama between 1941 and 1960. Led by Johnson T. Phyllis, Robert B. Tesh, Howard A. Christensen, Byron N. Chaniotis and Aristides Herrer research peaked in the next two decades, but shifted towards other areas such as *Leishmania* infections in the vectors, population dynamics, and interactions among parasites, vectors and hosts. These scientists and others put a great deal of effort into experimental and field work to disentangle the bionomics (i.e., the ecology and behavior) of *Lutzomyia* vector species and to identify the epidemiological determinants of the transmission cycle of ACL. During the next 30 years, research on ACL continued to focus generally on the same subjects but the rate of publication decreased gradually [Figure 2].

Collectively, the massive intellectual effort on ACL in Panama spanned roughly a century and resulted in approximately 100 peer-reviewed publications in international journals (Additional file 1: Table S1). These efforts stand as one of the greatest scientific contributions to the understanding of ACL epidemiology in Latin America. Recently, however, Panama has undergone significant changes in land use and human demography, and the epidemiology of ACL is thought to have changed considerably [14,17-24]. Surprisingly, few attempts have been made to summarize existing information about ACL in the country, despite its importance for predicting and controlling future epidemics [25]. In a review paper by Christensen *et al.,*[11], which dates back to 1983, the authors synthesized information on the ecology of ACL, sand flies, animal reservoirs and the available clinical data from endemic areas of Panama. However, their review included all *Lutzomyia* sand fly species, rather than focusing on the anthropophilics. If effective vector control measures are to be implemented in Panama, information about the ecology and behavior of epidemiologically important *Lutzomyia* species needs to be synthesized [23-25]. Herein, we summarize the body of literature concerning the epidemiology of ACL and the bionomics of *Lutzomyia* sand flies in Panama, including information that was published after Christensen *et al.,*[11], putting special emphasis on the most common anthropophilic species. This information will improve our understanding of potential changes in the transmission dynamics of this infection and contribute to the development of control strategies to limit *Leishmania* expansion across the country.

## Review

### Epidemiology

**Figure 3:**
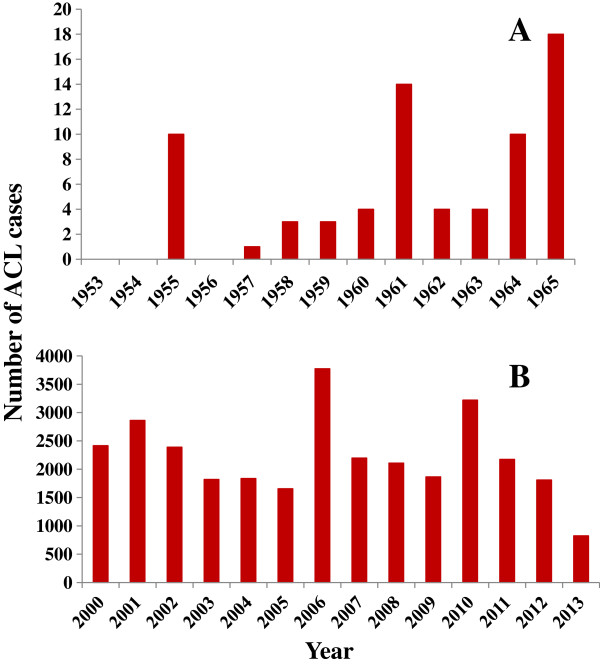
**The graphs show the chronological distribution of American Cutaneous Leishmaniasis (ACL) cases in Panama in two different periods of time: (A) represents the number of clinical cases recorded from 1953 to 1965 and (B) from 2000 to 2013.** Information in **A** and **B** was obtained directly from Walton *et al.,* (1968) and from the department of epidemiology of the Panamanian Ministry of Health (MINSA - as it is abbreviated in Spanish), respectively.

Historically, cases of ACL in Panama have fluctuated erratically, perhaps reflecting epidemic periods. However, these fluctuations may also be due in part to considerable underreporting because this disease has never been systematically monitored across the country [26]. Recently, some researchers have suggested a rising trend in the number of ACL cases in Panama, and attributed this to a lack of medical treatment, increased human migration into *Leishmaniasis* endemic areas, and/or to ecological changes triggering vector adaptation to human settlements in environmentally altered forest ecosystems [14,17-24]. Prior work in Panama though had also described a tendency toward increasing incidence of ACL, perhaps due to reasons different than those recently anticipated [4-9,11,12]. The scenario proposed earlier begins with a rapid increase in the number of clinical cases when susceptible human populations invade pristine tropical forest and modify the surroundings for settlements, thus increasing contact with the vectors and reservoirs of *Leishmania* parasites [Figure 1]. However, subsequent landscape alteration triggers demographic changes in wild animals, occasionally resulting in major reservoirs of *Leishmania* migrating out of the territory, and as a result, ACL cases drop off considerably. Finally, the disease disappears when sand fly vectors perish due to the lack of appropriate breeding conditions in the increasingly urban landscape [4-9,11,12]. This view is well-accepted as Panama has always been under similar environmental pressures (e.g., deforestation), and therefore, the recently-reported ACL increase is most likely an artifact of enhanced surveillance due to improved diagnostic tools and better access to health care [18,22,27] [Figure 3]. Alternatively, the increasing trend in the number of ACL cases could be the result of greater human-vector contact owing to higher rates of deforestation and human population growth across areas of old-growth forest in Panama during the last 30 years [28].

#### *Leishmania* transmission cycles

Many more studies have been undertaken on the vectors of ACL in Panama than on *Leishmania* parasites [Figure 2]. To date, seven *Leishmania* species have been reported in the country, but only three have been isolated from humans: *Leishmania* (*Viannia*) *panamensis*, *Le.* (*Viannia*) *colombiensis* and *Le.* (*Leishmania*) *amozanensis*, with the former being by far the most predominant disease agent [2,6,10-12,18,29-33]. Furthermore, three other species *Le.* (*Leishmania*) *aristidesi*, *Le.* (*Leishmania*) *hertigi* and *Le.* (*Viannia*) *naiffi* have been detected either from animal hosts (e.g., *Le.* (*L*) *aristidesi* was isolated from *Oryzomys capito*, *Agouti paca, Marmosa robinsoni* and *Proechimys semispinosus* and *Le.* (*L*) *hertigi* from *Coendou rothschildi*) or from wild-caught *Lutzomyia* sand flies (e.g., *Le.* (*V*) *naiffi* was isolated from pools of *Lu. trapidoi* and *Lu. gomezi*), of which only the latter is pathogenic to humans [1,11,34-36].

The enzootic cycles of *Le.* (*V*) *panamensis* and *Le.* (*L*) *aristidesi* are best known in Panama, but ecological knowledge about the transmission dynamics of other species remains incomplete [29,34,37-40]. These two parasites appear to utilize different sand fly vectors and vertebrate reservoirs, and their transmission dynamics seem to be governed by a different set of epidemiological factors. *Leishmania* (*Viannia*) *panamensis* is certainly transmitted by several species of *Lutzomyia* sand flies across the country, whereas *Le.* (*L*) *aristidesi* appears limited to eastern Panama, where it seems to be transmitted by a single sand fly species. *Lutzomyia trapidoi* and *Lu. panamensis*, the proven vectors of *Le.* (*V*) *panamensis* in Panama and Colombia as well as other suspected vectors such as *Lu. gomezi*, *Lu. ylephiletor*, *Lu. sanguinaria* and *Lu. carrerai thula* are very abundant and highly aggressive anthropophilic species. They feed opportunistically, primarily in the understory, but can do so in the forest canopy as well [12,41,42]. In contrast, *Lutzomyia olmeca bicolor*, the likely vector of *Le.* (*L*) *aristidesi*, is considered a rare subspecies that feeds almost exclusively on rodents and is most active at ground level [37,43]. Despite several animal species being found infected in nature with *Le.* (*V*) *panamensis* and *Le.* (*L*) *aristidesi*, studies aimed at identifying the reservoirs of these two parasites have indicated that they are largely restricted to one arboreal and one terrestrial mammal species, respectively [29,37,38,44-47]. This may explain why transmission intensity does not appear to be a function of vector density, but rather seems to be related to the density of the animal reservoirs [45,47]. For example, high transmission rates of *Le.* (*V*) *panamensis* may occur in areas of old-growth forest with high population densities of the two-toe sloth, *Choloepus hoffmanni*. Similarly, elevated densities of the rice-rat, *Oryzomys capito*, in areas of secondary forest and agricultural land seem to favor high transmission rates of *Le.* (*L*) *aristidesi*[37,38,44,48]. Other animals, including *Didelphis marsupialis* (Opossum) and *Proechimys semispinosus* (Spiny rat), may serve as secondary reservoirs for these parasites in Panama, but their epidemiological roles have not been fully investigated [44,46]. A likely reason why ACL transmission does not occur on domestic environments in endemic areas of Panama is that dogs and humans appear to be dead end-hosts for *Le.* (*V*) *panamensis*[4,11,44,49].

### Summary of research on vector taxonomy

Research on sand fly taxonomy represents less than 20% of the ACL studies undertaken in Panama, yet these efforts are essential for understanding the role of *Lutzomyia* species as vectors of this infection [Figure 2]. Raymond C. Shannon described the first species of sand fly from the country in 1926, at which time there were only nine species formally described from South America. *Phlebotomus panamensis* was first collected by Shannon with a sweeping net near the roots of a Cuipo tree in Cano Saddle, Gatun Lake and later again in Portobelo on the Atlantic side of the Isthmus. By 1961, Fairchild and Hertig had described more than 20 species of sand flies from numerous locales across Panama, although most originated from the Former Panama Canal Zone [50-64]. These taxonomic descriptions included specimens from other non blood-sucking genera of Psychodidae (e.g., *Warileya* and *Hertigia*), but most of them dealt with the adult stage of members of the genus *Phlebotomus* (Herein = *Lutzomyia*) [65] (Additional file 2: Table S2).

Three taxonomic papers written by Fairchild and Hertig were of particular importance for understanding the transmission cycle of ACL in Panama: the morphological separation of *Lu. gomezi* and *Lu. trinidadensis*[52]; the description of *Lu. trapidoi* and *Lu. ylephiletor* (also known as *Lu. ylepiletrix*) [55]; and the re-description of *Lu. panamensis*[56]. The first two taxa are morphologically similar, very abundant species that often co-occur geographically, but only *Lu. gomezi* is considered an important vector of ACL in Panama because it feeds regularly on humans. Moreover, *Lu. trapidoi*, *Lu. ylephiletor* and *Lu. panamensis* are among the most prevalent man-biters as well as proven ACL vectors in Panama and in other Neotropical countries [2,6,18,33,41,42,66-68]. Additional systematic work on sand flies from Panama described two new taxa one of which was *Lu. olmeca bicolor*, and added new records for other species described elsewhere, for a total of 74 morphologically characterized taxa for the country [69,70].

#### Taxonomic markers

Fairchild and Hertig published several morphological keys of *Lutzomyia* species from Panama, including epidemiologically relevant *Leishmania* vectors, as well as numerous other species from other parts of Central America [50,55-61,63,71]. This initial taxonomic work emphasized the female cibarium and the male genitalia as the most valuable morphological characters for distinguishing species. These structures contain a suit of characters that make species discrimination in both sexes accurate and feasible in a short period of time [63]. In contrast, morphometric analyses using venational characters (e.g., overall wing and vein lengths) and/or the palpal formula (e.g., listing the palpus segments in order of increasing length) have been shown to have little systematic value because these characters vary considerably among members of well-recognized morphological species and also among individuals of the same taxon [51,52,54,55,57,58,63,70], [72].

Taxonomic studies of the immature stages of sand flies began formally in Panama with the doctoral dissertation of Wilford J. Hanson in 1955, which was later published in the *Annals of the Entomological Society of America*[73]. Contrary to the approach used in adult taxonomy, which focused on internal structures, identification of the immature stages depended more on the Chaetotaxy (e.g., the distribution of setae in the insect body). Setae at the head capsule, thorax and abdomen vary in number, position, length and shape and are very important for species determination. These sensorial structures are thought to be serially homologous between adjacent segments, and so are comparable among the prothrorax, mesothorax and metathorax [73,74]. More recent taxonomic work using molecular markers supports the validity of 16 species of sand flies from central Panama including *Lu. trapidoi*, *Lu. panamensis*, *Lu. ylephiletor*, *Lu. carrerai thula* and *Lu. sanguinaria*, but suggests lineage divergence in *Lu. gomezi*[33]. The authors of this study hypothesized that *Lu. gomezi* is a cryptic species complex based on phylogenetic analyses using partial DNA sequences from the Folmer or “Barcode” region of the mitochondrial *CO1* gene. This finding is of particular epidemiological significance because morphologically identified *Lu. gomezi* was found infected with *Le.* (*V*) *naiffi,* at an infection rate of 23%. This pathogen causes cutaneous Leishmaniasis in South America and had never been reported in Panama previously [33]. Although there seems to be a high level of congruence between morphology and DNA Barcoding based taxonomy for *Lutzomyia* species in Panama, there is still a need to corroborate species boundaries using samples from across the entire country [75].

### Summary of studies on the bionomics of anthropophilic *Lutzomyia* species

#### Larval breeding habitats

Research on sand fly larval ecology in Panama has tackled aspects such as species habitat associations and environmental factors affecting population dynamics [11,45,63,73,74,76-79]. These studies relied on either direct observations or sampling techniques and resulted in a series of valuable publications that increased understanding of the biology and ecology of these insects. Hanson [73] collected fourth instar larvae of *Lu. trapidoi*, *Lu. panamensis*, *Lu. ylephiletor* and *Lu. carrerai thula* in dead (decaying) leaves from the forest floor of well-shaded areas [Figure 1]. His research demonstrated that larvae of these species tend to gather on moist, decaying areas on the upper and lower surfaces of leaves [73,74]. Furthermore, both females and males of *Lu. trapidoi*, *Lu. panamensis*, *Lu. ylephiletor* and *Lu. carrerai thula* were encountered resting in the same habitat during the day, suggesting that forest leaf-litter may not only serve as emergence and resting sites, but also as ovipositon and mating grounds for these taxa [11].

Studies by Rutledge and Ellenwood [49] used soil emergence traps in the forest floor of Gamboa, central Panama, to test for larval-habitat associations in sand flies. *Lutzomyia trapidoi* was most abundant on open forest floor, whereas *Lu. panamensis*, *Lu. gomezi* and *Lu. carrerai thula* were collected regularly, but in significantly lower numbers. Furthermore, the micro-spatial distribution of anthropophilic species appeared to vary with plant species composition, which ultimately seems to determine the size of the population in a given area. For example, larvae of *Lu. panamensis* and *Lu. gomezi* tended to be more abundant in association with large trees of the genus *Anacardium* (Anacardiaceae) while larvae of *Lu. trapidoi* were usually more prevalent in association with large lianas in the genera *Ourouparia* and *Sabicea* (Rubiaceae) [77]. Additionally, hydrological and physiographic factors such as soil moisture, erosion, percentage of shade and the depth of the forest leaf-litter affect soil movement and stability, and thus can also influence species distribution in the forest floor. For instance, larvae of *Lu. panamensis* and *Lu. gomezi* were more abundant in hilltops where forest leaf-litter was more stable, whereas *Lu. trapidoi* colonized hillside and streamside regions where unstable alluvial deposits were more common [76].

#### Adult resting sites and vertical stratification

Diurnal resting sites for anthropophilic *Lutzomyia* species in forest environments of Panama were described by Christensen *et al.,*[6,11], Christensen and Vasquez [68], and Chaniotis *et al.,*[80-82]. On the whole, this work indicated that tree hollows and animal burrows were the most productive resting habitats in terms of species richness, but suggested that tree buttresses and forest leaf-litter were more important from the epidemiological stand point [Figure 1]. This is because the latter were the most conspicuous resting habitats in the forest, and were also preferred by anthropophilic species. These studies also indicated that *Lu. trapidoi*, *Lu. panamensis* and *Lu. gomezi* were more abundant in forest leaf-litter, whereas *Lu. ylephiletor* and *Lu. carrerai thula* were more prevalent in tree trunks and in green plants, respectively [11,81]. Moreover, *Lu. ylephiletor* dominated tree buttresses at six different ACL sylvatic foci across the country, where it represented roughly 43% of the total sample of resting sand flies [12,68].

Studies of vertical stratification of sand flies in Panama were conducted in forest environments using both resting and blood-seeking samples as well as various collecting methods (e.g., human and animal baits, light traps and resting collections) [11,40,49,68,80-83]. In general, the results suggested that there is some degree of vertical partitioning of the niche because females of most species tend to cluster at the ground level, but few of them are active in the canopy. These behaviors are more obvious in *Lu. panamensis*, *Lu. gomezi*, *Lu. ylephiletor* and *Lu. carrerai thula*, whose populations are usually more numerous a few meters off the ground. In contrast, *Lu. trapidoi* and *Lu. sanguinaria* are more abundant at higher elevations (but see also [41]). *Lutzomyia trapidoi* might be the only proven acrodendrophilic species in the country, because it has been found 18 meters above the ground, both resting and blood-seeking, whereas *Lu. sanguinaria* has only been collected in light traps at this elevation [11,84]. Nevertheless, the extent of vertical stratification also varied geographically. For example, *Lu. panamensis* is considered a ground-level feeder in central Panama, but it was the most abundant species in the canopy in one study carried out in eastern Panama [49]. Similarly, *Lu. gomezi* and *Lu. sanguinaria* have been collected in great numbers in the forest canopy in Bocas Del Toro, despite being considered understory species in most studies carried out in central Panama [11,41]. Moreover, in some circumstances *Lu. trapidoi* has been the most abundant species at the understory regardless of which collecting method was employed. Therefore, rather than clustering at different vertical strata, anthropophilic sand fly species may move freely between the canopy and the understory seeking their preferred vertebrate hosts; and while doing so, they may also take blood from alternative hosts depending on their relative abundance [Figure 1]. This might explain why *Lu. trapidoi*, *Lu. panamensis*, *Lu. gomezi* and *Lu. sanguinaria* exhibit different host-feeding preferences at different vertical strata in different sites (See next section).

#### Biting activity and vector-host interactions

Chaniotis *et al.,*[80] investigated the daily biting activity of anthropophilic *Lutzomyia* species in central Panama. In agreement with similar studies from other regions of the country, they found that roughly 80% of the female biting takes place during crepuscular periods, mainly from 6:00 to 9:00 pm [11,49,66,67,85]. This pattern remained the same for most anthropophilic species, despite differences in density across vertical strata and climatic season, and seemed to be primarily associated with a reduction in light intensity [12]. However, *Lu. carrerai thula*, a dominant subspecies in the understory, was unaffected by light intensity and exhibited higher daytime biting rates, being more active at low temperature and high humidity [11,12,68]. Furthermore, studies on the horizontal movements of *Lutzomyia* have reported a major reduction in the biting rate of *Lu. panamensis*, *Lu. trapidoi* and *Lu. carrerai thula* in collections made 50 meters away from areas of old-growth tropical forest. Collectively these findings suggest that the highest risk of ACL transmission in endemic areas of Panama occurs early at night and inside the forest, but diurnal transmission might also occur where *Lu. carrerai thula* is prevalent. The role of this taxon as a vector of *Le.* (*V*) *panamensis* in the country has not been confirmed though [11,83].

Research on *Lutzomyia* host interactions began in Panama with the pioneering work of Tesh *et al.,*[66,67], who employed the precipitin test and nine Order-specific mammalian antisera to provide the first assessment of *Lutzomyia* feeding preferences in the Americas. Their results suggested that some anthropophilic species are highly specific at the Order level and prefer to feed upon a single mammalian Order (e.g., 73% of *Lu. carrerai thula* fed on Edentates and 100% of *Lu. sanguinaria* fed on Primates). However, the two most abundant species, *Lu. trapidoi* and *Lu. ylephiletor*, exhibited opportunistic feeding behavior, shifting host preferences across sites according to changes in the relative abundance of the mammalian fauna. Interestingly, *Lu. trapidoi* and *Lu. ylephiletor* shifted blood choices from Edentates to rodents and marsupials between undisturbed forest at El Limbo Field Station, in central Panama, and human-altered forest ecosystems at Finca Montenegro and Finca el Valle, respectively. This was the first evidence in Panama that deforestation could alter the feeding preferences of *Lutzomyia* sand fly species by triggering host community changes and/or host species decline. Results from Tesh *et al.,*[66,67] were in disagreement with those from Christensen and Vasquez [69], who studied *Lutzomyia* host-feeding sources across the country, including localities from western and central-eastern Panama. While both *Lu. trapidoi* and *Lu. ylephiletor* favored Edentates over other mammalian Orders in Tesh *et al.,*[66,67], Christensen and Vasquez [69] reported that *Lu. ylephiletor* fed on more than 20 different families of mammals, birds, reptiles and even amphibians, whereas *Lu. trapidoi* fed almost exclusively on sloths (e.g., *Choloepus hoffmanni*). The discrepancies between studies may be attributed to a lower resolution in the study by Tesh *et al.,*[66], which did not determine host taxonomic identity beyond the class level, and also due to potential sampling biases as numerous sentinel monkeys and rodents were set up in the study area [67]. Alternatively, the larger number of sampling sites included in the study by Christensen and Vasquez [68] may indicate that *Lu. ylephiletor* is more of a catholic feeder than *Lu. trapidoi*.

Evidence from Panamanian studies suggests that there are six anthropophilic species of *Lutzomyia* in ACL endemic areas of Panama: *Lu. trapidoi*, *Lu. panamensis*, *Lu. ylephiletor*, *Lu. gomezi*, *Lu. sanguinaria* and *Lu. carrerai thula*[12,44,66-68,80,81,86-89]. In general, these species are opportunistic, which makes them potential transmitters of pathogens across a broad range of wildlife, domesticated animals and humans [84,90]. Nevertheless, as pointed out before, under certain circumstances some species can also be host-specific. For instance, *Lu. sanguinaria* has been found to feed upon five different mammalian Orders in Sasardi Kuna Yala, eastern Panama, but exhibited a strong preference for dogs in central Panama [11,49]. These studies further support the view that *Lutzomyia* host trophic interactions (e.g., host-specific vs. opportunistic, and canopy vs. understory feeding) are ultimately determined by the richness and relative abundances of animal host species in forest environments of Panama, which in turn are affected by the degree of human alteration in these places (e.g., deforestation).

#### Seasonal trends

Evidence for sand fly seasonality in Panama comes from both larval and adult studies, but results from these investigations are not readily comparable due to differences in sampling approaches and ecological factors inherent to each developmental stage. Nevertheless, the two approaches have reached similar conclusions [11,49,87]. Chaniotis *et al.,*[79,84] studied the seasonality of Phlebotominae sand flies at El Limbo Field Station, in central Panama. The results demonstrated that most anthropophilic species peak in density during the wet season, although populations of *Lu. panamensis* and *Lu. carrerai thula* increased in the early wet season between June and August, whereas *Lu. trapidoi*, *Lu. ylephiletor* and *Lu. sanguinaria* increased in October and November during the late wet season (but see also [47,87,88]). Furthermore, wet-adapted species decreased significantly during the dry season; disappearing almost entirely from the study area, while *Lu. gomezi*, a dry-adapted species, was more abundant at this time of the year. Studies by Chaniotis *et al.,*[80,86] also indicated that overall sand fly seasonal trends (which included the anthopophilic species) did not vary according to biotope (old-growth forest vs. secondary growth forest), vertical strata (canopy vs. understory) or sex (female vs. male). Abundances were mostly influenced by the amount and distributional pattern of rainfall, as there was little variation in monthly mean temperature and relative humidity [86]. Similarly, studies on the patterns of adult emergence in the forest soils of central Panama have confirmed that anthropophilic sand fly species are highly seasonal, with cyclical trends most evident in *Lu. trapidoi* and *Lu. gomezi*. Newly emerged populations of these two species peaked in density in different periods of the year: the former being more abundant during the rainy season, from May to September, and the latter increasing in numbers from January to April [45]. Overall, these seasonal patterns may be associated with higher transmission rates of ACL during the rainy season in Panama, likely due to increased *Leishmania* infection rates in vector populations around this time of the year. However, as pointed out earlier, the distribution and prevalence of ACL in Panama suggests that vector density has little influence on transmission intensity, which could be even higher in sites where zoophilic sand fly species dominate over anthropophilics [45,88].

### Vector control

Studies on sand fly control are very limited in Panama and account for less than five scientific publications during the entire history of ACL research in this country [12,23,24,91] [Figure 2]. This may be in part due to the nature of this non-fatal and chronic zoonotic disease, which affects people in remote rural areas rather than in urban centres [26]. It may also reflect the complicated and impractical task of controlling sand fly populations in forest environments [49]. Early studies of vector control in Panama evaluated three methods of personal protection [91]. Researchers found that skin applications with N,N-diethyl-meta-toluamide (DEET) conferred good protection against the bites of *Lu. panamensis*, *Lu. gomezi* and *Lu. sanguinaria*. However, this protection lasted only for a few hours, whereas DEET-treated net jackets offered protection for up to two weeks. In contrast, permethrin-treated clothing did not offer protection against *Lutzomyia* bites. It was concluded that full protection could only be achieved by combining the use of DEET-treated net jackets with DEET skin applications on those areas that are not covered by the jacket [91].

Insecticide control trials with 95% technical grade malathion using Ultra Low Volume (ULV) and 2% malathion Emulsifiable Concentrate (EC) in hand-operated sprayers were carried out by Chaniotis *et al.,*[12] to attempt to control sand fly populations in a sylvatic focus of ACL on the Atlantic coast of central Panama. The authors applied insecticide around *Lutzomyia* resting sites to prevent ACL transmission to military personnel, and assessed the efficacy of this treatment by collecting blood-seeking and resting females near tree trunks after treatment. They found that sand fly density was reduced by 52.8% and 44.7% by ULV and EC applications, respectively, in comparison with the pretreatment period. However, differences were only statistically significant for the latter. More recent studies using insecticide thermal fogging in a hyperendemic focus of ACL in central Panama reported a decrease in the *Lutzomyia* species richness between deltamethrin-treated and untreated houses, although anthropophilic species were still present after treatment. The relative abundance of *Lu. gomezi* and *Lu. panamensis* was reduced by roughly 50% in deltamethrin-treated houses, but the population of *Lu. trapidoi* increased by 5%. The authors hypothesized that sand fly re-infestation was facilitated by poor housing quality, since unsuitable building materials would either provide additional resting sites for sand flies or hamper insecticide efficacy by reducing its active lifespan [23,24]. Together these findings reflect the short-lasting nature of these insecticide control measures, which do not affect the immature stages of sand flies in the forest. However, these observations do highlight the importance of evaluating the effectiveness of insect repellents and insecticide treatment, in order to successfully integrate strategies for *Lutzomyia* control in Panama. Results from these studies also emphasize the need for adopting a community based approach to better understand the role of anthropophilic *Lutzomyia* species on ACL transmission dynamics across the country [23,24]. Considerably more scientific work on vector control is required in Panama, including the need to follow up on studies about the presence of the bacterium *Wolbachia pipientis* in pools of *Lu. trapidoi* as a potential biocontrol strategy to mitigate *Lutzomyia* populations [33].

## Conclusion

The last review paper published about ACL research in Panama was written 30 years ago [11]. Since then the country has undergone significant changes in landscape and human demography, and more people are commonly in contact today with forest environments where the sylvatic focus of ACL takes place. As a result, more clinical cases are being reported in local human populations as well as in international visitors. This increase in clinical cases could also be due to underreporting in the past or improved *Leishmania* diagnostic tools and better access to health care at present. Advanced research about ACL epidemiology and *Lutzomyia* bionomics is still needed in Panama. These efforts will be greatly strengthened by the integration of modern technologies such as bioinformatics, modeling, and genomic markers to investigate population genetics and molecular taxonomy of anthropophilic *Lutzomyia* species. In addition, more sophisticated analyses using geographic information systems (GIS) and ecological niche modeling (ENM) techniques on vector species will be required to predict ACL transmission risk across the country. Finally, future research about ACL in Panama will benefit from adopting a more holistic approach, where integrated vector control strategies are planned based on information generated from scientific research. We hope that our review will contribute to this goal as it provides a synopsis of taxonomic and bionomic information of epidemiologically relevant *Lutzomyia* species in the country. This information, along with knowledge about the impact of human landscape modifications on ACL epidemiology, will help to predict and control future epidemics.

## Abbreviations

ACL: American Cutaneous Leishmaniasis; DNA: Deoxyribonucleic acid; CO1: Cytochrome C oxidase one; DEET: N,N-diethyl-meta-toluamide; ULV: Ultra low volume; EC: Emulsifiable concentrate; GIS: Geographic information system; ENM: Ecological niche modeling.

## Competing interests

The authors declare that they have no competing interests.

## Authors’ contributions

LCD and JRL wrote the review; both authors read and approved the final version of the manuscript.

## Supplementary Material

Additional file 1: Table S1 Body of literature concerning the epidemiology of American Cutaneous Leishmaniasis (ACL) and the bionomics of *Lutzomyia* sand flies in Panama. Click here for file

Additional file 2: Table S2 Record of taxonomic studies of *Lutzomyia* sand fly species from Panama. Click here for file
