# Supplementary material for: American Cutaneous Leishmaniasis in Panama: a historical review of entomological studies on anthropophilic Lutzomyia sand fly species
Source: Parasit Vectors. 2014 May 11;7:218. doi: 10.1186/1756-3305-7-218 (PMC4026118; doi:10.1186/1756-3305-7-218)
Supplement: Additional file 1: Table S1 — Body of literature concerning the epidemiology of American Cutaneous Leishmaniasis (ACL) and the bionomics of Lutzomyia sand flies in Panama. [file 1756-3305-7-218-S1.docx]

| **Publication Title** | **Subject** | **Reference** | **Year** |
| --- | --- | --- | --- |
| Cutaneous Leishmaniasis: Review | ACL Epidemiology | [1]* | 2007 |
| Phlebotomine sanflies and the spreading of Leishmaniases and other diseases of public  health concern | ACL Epidemiology | [2]* | 2013 |
| Biology of Phlebotomine sand flies as vectors of disease agents | Vector Bionomic | [3]* | 2013 |
| Epidemiological patterns of Cutaneous Leishmaniasis in Panama, III. Endemic persistence  of the disease | ACL Epidemiology | [4] | 1976 |
| Epidemiological patterns of Cutaneous Leishmaniasis in Panama. II. Incidental occurrence  of cases in no-endemic settlements | ACL Epidemiology | [5] | 1976 |
| Short report epidemiologic studies on Cutaneous Leishmaniasis in eastern Panama | ACL Epidemiology | [6] | 1999 |
| Cutaneous Leishmaniasis on the Isthmus of Panama | ACL Epidemiology | [7] | 1944 |
| American Cutaneous Leishmaniasis: report of 12 cases from canal zone | ACL Epidemiology | [8] | 1948 |
| Epidemiological patterns of Cutaneous Leishmaniasis in Panama. I.  Epidemics among small groups of settlers | ACL Epidemiology | [9] | 1976 |
| Cutaneous Leishmaniasis associated with jungle training | ACL Epidemiology | [10] | 1980 |
| The ecology of Cutaneous Leishmaniasis in the Republic of Panama | ACL Epidemiology | [11] | 1983 |
| A pilot study to control Phlebotomine sand flies (Diptera: Psychodidae)  in a Neotropical rain forest | Vector Control | [12] | 1982 |
| Acquisition, development and treatment of Panamanian Cutaneous Leishmaniasis: a case history | ACL Epidemiology | [13] | 1988 |
| History of US Military contributions to the study of parasitic diseases | ACL Epidemiology | [14] | 2005 |
| Leishmaniasis in the U.S. military Canal Zone | Vector Bionomic | [15] | 1968 |
| The occurrence of *Phlebotomus* in Panama | ACL Epidemiology | [16] | 1926 |
| Leishmaniasis in the Caribbean Islands: A review | ACL Epidemiology | [17]* | 1992 |
| Molecular epidemiology of American Tegumentary Leishmaniasis in Panama | ACL Epidemiology | [18] | 2009 |
| Relación entre la composición de especies del Género de *Lutzomyia franca*  (Diptera: Psychodidae: Phlebotominae) y los diferentes tipos de bosques en Panamá | Vector Bionomic | [19] | 2008 |
| Anthropogenic influence on the distribution, abundance and diversity of sandfly species  (Diptera: Psychodidae: Phlebotominae),vectors of Cutaneous Leishmaniasis in Panama | Vector Bionomic | [20] | 2011 |
| Amplified fragment length polymorphisms reveals high intraspecific variability in field isolates  of *Leishmania panamensis* | Leishmania Research | [21] | 2011 |
| Clinical Cutaneous Leishmaniasis rates are associated with household *Lutzomyia gomezi*,  *Lu. panamensis* and *Lu. trapidoi* abundance in Trinidad de Las Minas, western Panama | ACL Epidemiology | [22] | 2013 |
| Changes in Phlebotomine sand fly species composition following insecticide thermal  fogging in a rural setting of western Panama | Vector Control | [23] | 2013 |
| Leishmaniasis sand fly vector density reduction is less marked in destitute housing after  insecticide thermal fogging | Vector Control | [24] | 2013 |
| A review of preventative methods against human Leishmaniasis infection | ACL Epidemiology | [25] | 2013 |
| Leishmaniasis worldwide and global estimates of its incidence | ACL Epidemiology | [26]* | 2012 |
| Evaluation of PCR for Cutaneous Leishmaniasis diagnosis and species identification  using filter paper samples in Panama, Central America | Leishmania Research | [27] | 2012 |
| Historical, demographic and economic correlates of land-use change in the Republic of Panama | Leishmania Research | [28] | 2008 |
| Detection of *Leishmania brazilienzis* by xenodiagnosis | Leishmania Research | [29] | 1972 |
| Estudios sobre la epidemiología de la Leishmaniasis en la parte occidental  de la República de Panamá | ACL Epidemiology | [30] | 1990 |
| Cutaneous leishmaniasis caused by *Leishmania mexicana amazonensis* in Panama | ACL Epidemiology | [31] | 1988 |
| Characterization of *Leishmania colombiensis* sp. (Kinetoplastida: Trypanosomatidae),  a new parasite infecting humans, animals and Phlebotominae sand flies in Colombia and Panama | Leishmania Research | [32] | 1991 |
| *Leishmania braziliensis* isolated from sloths in Panama | Leishmania Research | [33] | 1969 |
| *Leishmania hertigi* sp. N. from the tropical porcupine, *Coendou rothschildi* Thomas | ACL Epidemiology | [34] | 1971 |
| Enzootic Cutaneous Leishmaniasis in eastern Panama. I. Investigation of the infection  among forest mammals | ACL Epidemiology | [35] | 1971 |
| *Lutzomyia* sandfly diveristy and rates of infection by *Wolbachia* and an exotic  *Leishmania* species in Barro Colorado Island , Panama | Vector Bionomic | [36] | 2010 |
| Reservoir host of Cutaneous Leishmaniasis among Panamanian forest mammals | ACL Epidemiology | [37] | 1973 |
| Development of a Panamanian strain of *Leishmania mexicana* in co-indigenous  *Lutzomyia sanguinaria* and *Lu. gomezi* (Diptera Psychodidae) | Leishmania Research | [38] | 1980 |
| Leishmania braziliensis in the Panamanian two-toed sloth, *Choloepus hoffmani* | ACL Epidemiology | [39] | 1980 |
| Mucocutaneous Leishmaniasis in Panama. Etiologic agent, epidemiologic and clinical aspects | ACL Epidemiology | [40] | 1989 |
| Natural infection of Leptomoned flagellates in Panamanian P*hlebotomus* sandflies | Vector Bionomic | [41] | 1963 |
| *Leishmania braziliensis*s. lat. isolated from *Lutzomyia panamensis* in Panama | Vector Bionomic | [42] | 1969 |
| Attractiveness of sentinel animals to vectors of Leishmaniasis in Panama | Vector Taxonomy | [43] | 1973 |
| Field studies on the feeding habits and diurnal shelters of some *Phlebotomus*  sandflies (Diptera: Psychodidae) in Panama | Vector Bionomic | [44] | 1966 |
| Production of Phlebotomine sandflies on the open forest floor in Panama: Hydrologic  and physiographic relations | Vector Bionomic | [45] | 1975 |
| Infrequency of gross lesions among Panamanian forest animals with Cutaneous Leishmaniasis | Leishmania Research | [46] | 1975 |
| A transect study of sand fly populations in Panama (Diptera: Psychodidae) | Vector Bionomic | [47] | 1976 |
| Enzootic Cutaneous Leishmaniasis in Eastern Panama. II. Entomological investigations | ACL Epidemiology | [48] | 1972 |
| Experimental infection of Panamanian *Phlebotomus* sandflies with *Leishmania* | Leishmania Research | [49] | 1963 |
| Notes on the *Phlebotomus* of Panama. I The subgenus *Brumptomyia* Franca and Parrot 1921 | Vector Taxonomy | [50] | 1947 |
| Notes on the *Phlebotomus* of Panama (Diptera: Psychodidae) II. Description  of three new species | Vector Taxonomy | [51] | 1947 |
| Notes on the *Phlebotomous* of Panama (Diptera, Psychodidae). III. *Phlebotomus cruciatus*  Coq. *trinidadensis* Newst. and *gomezi* Nitz | Vector Taxonomy | [52] | 1948 |
| Notes on the *Phlebotomus* of Panama (Diptera: Psychodidae) IV. *Phlebotomus atroclavatus*, *P. chiapannensis* Damp and some related forms from the West Indies and Mexico | Vector Taxonomy | [53] | 1948 |
| Notes on the *Phlebotomus* of Panama (Diptera. Psychodidae) VI. *Phlebotomus Shannon*  Dyar and related species | Vector Taxonomy | [54] | 1950 |
| Notes on the *Phlebotomus* of Panama (Diptera, Psychodidae) VII. The subgenus  Shannoniana Pratt | Vector Taxonomy | [55] | 1950 |
| Notes on the P*hlebotomus* of Panama. IX. Descriptions of seven new species | Vector Taxonomy | [56] | 1952 |
| Notes on the *Phlebotomus*of Panama (Diptera: Psychodidae) X. *Phlebotomus aragoai,*  *P. barretoi* and two new species | Vector Taxonomy | [57] | 1953 |
| Notes on the *Phlebotomus* of Panama (Diptera, Psychodidae). XI. The male of *Phlebotomus cruciatus* Coq., and notes on related species | Vector Taxonomy | [58] | 1953 |
| Notes on the *Phlebotomus* of Panama (Diptera: Psychodidae) XII. The group Anthophorus,  with descriptions of four new species from Panama and Mexico | Vector Taxonomy | [59] | 1956 |
| Notes on the *Phlebotomus* of Panama XIII. The Vexator group, with descriptions of new  species from Panama and California | Vector Taxonomy | [60] | 1957 |
| Notes on the *Phlebotomus* of Panama (Diptera: Psychodidale) XIV. *Phlebotomus*  *vespertilionis* and related species | Vector Taxonomy | [61] | 1958 |
| Notes on the *Phlebotomus* of Panama (Diptera: Psychodidae) XV. Four apparently  new synonymies | Vector Taxonomy | [62] | 1958 |
| Notes on the *Phlebotomus* of Panama (Diptera, Psychodidae) XVI. Descriptions of new  and little-known species from Panama and Central America | Vector Taxonomy | [63] | 1961 |
| *Lutzomyia tintinabula* n. sp. (Diptera: Psychodidae) from Panama | Vector Taxonomy | [64] | 1971 |
| Notes on the *Phlebotomus* of Panama (Diptera: Psychodidae) VIII. Two new species of *Warileya* | Vector Taxonomy | [65] | 1951 |
| Natural host preference of Panamanian Phlebotominae sandflies as determined by precipitin test | Vector Bionomic | [66] | 1971 |
| Further studies on the natural host preferences of Panamanian Phlebotominae sandflies | Vector Bionomic | [67] | 1972 |
| The tree-buttress biotope: a pathobiocenose of *Leishmania braziliensi*s | ACL Epidemiology | [68] | 1982 |
| On *Lutzomyia flaviscutellata* (Mangabeira) and *Lutzomyia olmeca* (Vargas and Díaz-Najera) | Vector Bionomic | [69] | 1971 |
| Check list of the Phlebotomine sand flies (Diptera: Psychodidae) of Panama including two  species not previously reported | Vector Bionomic | [70] | 1972 |
| Use of external characters for rapid identification of Phlebotominae sandflies in vector studies | Vector Taxonomy | [71] | 1974 |
| Notes on the *Phlebotomus* of Panama (Diptera: Psychodidae) V. The second sternite as a taxonomic character | Vector Taxonomy | [72] | 1950 |
| The breeding places of *Phlebotomus* in Panama (Diptera: Psychodidae) | Vector Bionomic | [73] | 1961 |
| The immature stages of the subfamily Phlebotominae in Panama (Diptera: Psychodidae) | Vector Bionomic | [74] | 1968 |
| Phylogeography of the *Lutzomyia gomezi* (Diptera: Phlebotominae) on the Panama Isthmus | Vector Bionomic | [75] | 2014 |
| Production of Phlebotomine sandflies on the open forest floor in Panama: the species complement | Vector Bionomic | [76] | 1975 |
| Production of Phlebotomine sandflies on the open forest floor in Panama: phytologic and  edaphic relations | Vector Bionomic | [77] | 1975 |
| The rearing of *Phlebotomus* sandflies (Diptera: Psychodidae) II. Development and behaviour of Panamanian sandflies in laboratory culture | Vector Bionomic | [78] | 1961 |
| Biology of immature sandflies at the bases of trees in Panama | Vector Bionomic | [79] | 1972 |
| Daily and seasonal man biting activity of Phlebotominae sandflies in Panama | Vector Bionomic | [80] | 1971 |
| Diurnal resting sites of Phlebotominae sandflies in Panamanian tropical forest | Vector Bionomic | [81] | 1972 |
| Field studies on the feeding habits and diurnal shelters of some *Phlebotomus* sandflies (Diptera: Psychodidae) in Panama | Vector Bionomic | [82] | 1966 |
| Comparative flying and biting activity of Panamanian Phlebotomine sandflies in a mature forest and adjacent open space | Vector Bionomic | [83] | 1974 |
| Horizontal and vertical movements of Phlebotominae sandflies in a Panamanian rain forest | Vector Bionomic | [84] | 1974 |
| Improved trapping of Phlebotomine sand flies (Diptera: Psychodidae) in light traps supplemented with dry ice in a Neotropical rain forest | Vector Bionomic | [85] | 1983 |
| Natural populations dynamics of Phlebotominae sandflies in Panama | Vector Bionomic | [86] | 1971 |
| Panamanian *Lutzomyia* (Diptera: Psychodidae) host attraction profiles | Vector Bionomic | [87] | 1980 |
| An analysis of sand fly light trap collections in the Panama canal zone (Diptera: Psychodidae) | Vector Bionomic | [88] | 1975 |
| Studies of Phlebotominae sand flies using castor oil traps baited with Panamanian animals | Vector Bionomic | [89] | 1968 |
| Phlebotomine sand flies as vectors of vesiculoviruses: A review | Vector Bionomic | [90]* | 1991 |
| Evaluation of personal protection methods against Phlebotomine sand flies including vectors of Leishmaniasis in Panama | Leishmania Research | [91] | 1982 |
| Behaviour of Leishmania in Panamanian Phlebotominae sandflies fed on infected animals | Leishmania Research | Non-cited | 1970 |
| Growth pattern of *Leishmania* in Phlebotominae sandflies | Leishmania Research | Non-cited | 1969 |
| Studies on the immunology and serology of Leishmaniasis III. On the cross  immunity between Panamanian Cutaneous Leishmaniasis and *Leishmania mexicana* | Leishmania Research | Non-cited | 1976 |
| Efficacy and toxicity of Pentostam against Panamanian mucosal Leishmaniasis | Leishmania Research | Non-cited | 1991 |
| Efficacy of ketoconazole against *Leishmania braziliensis panamensis* Cutaneous Leishmaniasis | Leishmania Research | Non-cited | 1990 |
| Development evaluation of the immunologic response in patients with Cutaneous Leishmaniasis | Leishmania Research | Non-cited | 1990 |
| Hydrosoluble formazan XTT: Its application to natural products drug discovery for *Leishmania* | Leishmania Research | Non-cited | 2003 |
| Leptomonads of wild-caught Panamanian *Phlebotomus*: culture and animal inoculation | ACL Epidemiology | Non-cited | 1963 |
| Sugar feeding behavior of *Lutzomyia trapidoi* (Diptera: Psychodidae) under experimental conditions | Vector Bionomic | Non-cited | 1974 |
| Autogeny in Panamanian *Phlebotomus* sandflies (Diptera: Psychodidae) | Vector Bionomic | Non-cited | 1961 |
| Colonization of *Lutzomyia trinidadensis* and *L. vespertilionis* (Diptera: Psychodidae) | Vector Bionomic | Non-cited | 1972 |
| *Lutzomyia vespertillionis* (Diptera: Psychodidae): Potential vector of chiropteran trypanosomes in Panama | Vector Bionomic | Non-cited | 1975 |
| Pyloric armature of New World Phlebotominae sandflies (Diptera: Psychodidae) | Vector Bionomic | Non-cited | 1971 |
| Neotropical sand flies (Diptera: Psychodidae), invertebrate hosts of *Endotrypanum*  *schaudinni* (Kineloplastida: Trypanosomatidae) | Vector Bionomic | Non-cited | 1976 |
| Report of *Lutzomyia longipalpis* (Lutz & Neiva, 1912) (Diptera: Psychodidae: Phlebotominae)  in a Cutaneous Leishmaniasis endemic area of Panama | Vector Bionomic | Non-cited | 2011 |
| Leishmaniasis (Oriental Sore) of the nasal mucosa | ACL Epidemiology | Non-cited | 1913 |
| Oriental sore in Panama | ACL Epidemiology | Non-cited | 1910 |
| Autochthonous Oriental sore in Panama | ACL Epidemiology | Non-cited | 1910 |
| Oriental sore in Panama | ACL Epidemiology | Non-cited | 1911 |
| Oriental Sore | ACL Epidemiology | Non-cited | 1911 |
| An unusual type of Oriental Sore | ACL Epidemiology | Non-cited | 1910 |

ACL = American Cutaneous Leishmaniasis

* These references are not part of Figure 2 because these studies were not conducted in Panama

Non-cited references can be found in full and alphabetic order below this table

**List of non-cited references in Suppl. - Table 1**

Bates LB: **Leishmaniasis (Oriental Sore) of the nasal mucosa.** *Proceed Canal Zone, Med Assoc* 1913, **5**: 83-84.

Darling ST: **Oriental sore in Panama.** *Proceed Canal Zone, Med Assoc* 1910, **3**: 7-20.

Darling ST: **Autochthonous Oriental sore in Panama.** *Trans Soc Trop Med & Hyg* 1910, **4:** 60-63.

Darling ST: **Oriental sore in Panama.** *Arch Int Med* 1911, **7:**581-597.

Darling ST: **Oriental sore in Panama.** *The Jour Cut Diseases* 1911, **29:**617-627.

Chaniotis BN: **Sugar-feeding behavior of *Lutzomyia trapidoi* (Diptera: Psychodidae) under experimental conditions**. *J Med Entomol* 1974, **11**(1):73-79.

Christensen HA: **Colonization of *Lutzomyia trinidadensis* and *Lu. vespertilionis* (Diptera: Psychodidae)**. *Ann Ent Soc Amer* 1972, **65**:683-686.

Christensen HA, Herrer A: ***Lutzomyia vespertilionis* (Diptera: Psychodidae): Potential vector of chiropteran trypanosomes in Panama**. *J Med Entomol* 1975, **12**:477-478.

Christensen HA, Herrer A, Fairchild GB: **Pyloric armature of New World Phlebotomine sandflies (Diptera: Psychodidae)**. *J Med Entomol* 1971, **8**:116-119.

Christensen HA, Herrer A**: Neotropical sand flies (Diptera: Psychodidae), invertebrate hosts of *Endotrypanum schaudinni* (Kineloplastida: Trypanosomatidae)**. *J Med Entomol* 1976, **13**:299-303.

De Carreira PF, Suárez MO, Pascale JM, Sousa OE: **Development evaluation of the immunologic response in patients with Cutaneous Leishmaniasis**. *Rev Med Panama* 1990, **15**(2):119-126.

Herrick AB: **An unusual type of Oriental Sore.** *Proceed* *Canal Zone, Med Assoc* 1910, **3**: 21-25.

Hertig M, Johnson PT, McConnell E, Anderson JR, Ayala SC: **Growth pattern of *Leishmania* in Phlebotomine sandflies**. *Science* 1969, **165**(3900):1379-1381.

Johnson PT, Hertig M: **Behavior of *Leishmania* in Panamanian Phlebotomine sandflies fed on infected animals**. *Expe Parasitol* 1970, **27**(2):281-300.

Johnson PT: **Autogeny in Panamanian *Phlebotomus* Sandflies (Diptera: Psychodidae)**. *Ann Ent Soc Amer* 1961, **54**(1):116-118.

Lainson R, Shaw JJ: Studies on the immunology and serology of leishmaniasis III. **On the cross-immunity between Panamanian Cutaneous Leishmaniasis and *Leishmania mexicana* infection in man**. *Trans R Soc Trop Med Hyg* 1966, **60**(4):533-535.

McConnell E: **Leptomonads of wild-caught Panamanian *Phlebotomus*: Culture and animal inoculation**. *Expe Parasitol* 1963, **14**(1):123-128.

Sáenz RE, De Rodríguez CG, Johnson CM, Berman JD: **Efficacy and toxicity of Pentostam against Panamanian mucosal Leishmaniasis**. *Am J Trop Med Hyg* 1991, **44**(4):394-398.

Sáenz RE, Paz H, Berman JD: **Efficacy of Ketoconazole against *Leishmania braziliensis panamensis* Cutaneous Leishmaniasis**. *Amer J Med* 1990, **8**(2):147-155.

Valderrama A, García Tavares M, Andrade Filho JD: **Report of *Lutzomyia longipalpis* (Lutz & Neiva, 1912) (Diptera: Psychodidae: Phlebotominae) in a Cutaneous Leishmaniasis endemic area of Panama**. *Mem Inst Osw Cruz* 2011, **106**(8):1049-1051.

Williams C, Espinosa OA, Montenegro H, Cubilla L, Capson TL, Ortega-Barría E, Romero LI: **Hydrosoluble formazan XTT, its application to natural products Drug discovery for *Leishmania***. *J Microbiol Methods* 2003, **55**(3):813-816.
