# Supplementary material for: American Cutaneous Leishmaniasis in Panama: a historical review of entomological studies on anthropophilic Lutzomyia sand fly species
Source: Parasit Vectors. 2014 May 11;7:218. doi: 10.1186/1756-3305-7-218 (PMC4026118; doi:10.1186/1756-3305-7-218)
Supplement: Additional file 2: Table S2 — Record of taxonomic studies of Lutzomyia sand fly species from Panama. [file 1756-3305-7-218-S2.docx]

| **#** | **Sand Fly Species** | **Type Locality** | **Province** | **Collecting Method / Site** | **References** |
| --- | --- | --- | --- | --- | --- |
| 1 | *Phlebotomus panamensis* | Caño Saddle (CZ) | Panama | Sweeping with a net | [16] |
| 2 | *Warileya nigrosacculus sp. nov.* | Cerro Campana | Panama | Hollow trees | [65] |
| 3 | *Phlebotomus dysponetus sp. nov.* | Juan Mina (CZ) | Panama | Automatic fan type light trap | [56] |
| 4 | *Phlebotomus acanthobasis sp. nov.* | Gatuncillo (CZ) | Panama | Hollow tree containing bats | [56] |
| 5 | *Phlebotomus aclydiferus sp. nov.* | Mojinga Swamp (CZ) | Panama | Mosquito light trap | [56] |
| 6 | *Phlebotomus vexillarius sp. nov.* | Serranía Maje, Cerro Chucanti | Panama | Tree buttress | [56] |
| 7 | *Phlebotomus triramulus sp. nov.* | Rio del medio | Colon | Tree buttress | [56] |
| 8 | *Phlebotomus ylephilletor sp. nov.* | Finca Nievecita, Almirante | Bocas del Toro | Buttressed roots | [56] |
| 9 | *Phlebotomus trapidoi sp. nov.* | Almirante | Bocas del Toro | Buttressed roots | [56] |
| 10 | *Phlebotomus shannoni* Dyar | Caño Saddle (CZ) | Panama | Crevices between buttressed roots | [54] |
| 11 | *Phlebotomus volcanensis sp. nov.* | Finca Carinthia, Cerro Punta | Chiriqui | Hollow trees and buttresses | [54] |
| 12 | *Hertigia hertigi* | Rio Chico (CZ) | Panama | Rock crevice | Non-cited |
| 13 | *Phlebotomus vespertillionis sp. nov.* | Cerro Campana | Panama | Shallow cave with bats | [51] |
| 14 | *Phlebotomus vesiciferus sp. nov.* | Cruces Trail (CZ) | Panama | Large hollow tree with bats | [51] |
| 15 | *Phlebotomus bispinosus sp. nov.* | La Victoria, Cerro Jefe | Panama | Shannon - light trap | [55] |
| 16 | *Phlebotomus hartmanni n. sp.* | Cerro Campana | Panama | Mosquito light trap | [60] |
| 17 | *Phlebotomus sanguinarius n. sp.* | Almirante | Bocas del Toro | Mosquito light trap | [60] |
| 18 | *Phlebotomus hamatus sp. nov.* | Chilibrillo, Chilibre (CZ) | Panama | Crevice in limestone rock | [50] |
| 19 | *Phlebotomus galindoi sp. nov.* | Boquete | Chiriqui | Sweeping with a net | [50] |
| 20 | *Phlebotomus odax* | Almirante | Bocas del Toro | Mosquito light trap | [63] |
| 21 | *Phlebotomus oresbius* | Santa Clara, Volcan | Chiriqui | Tree buttresses | [63] |
| 22 | *Phlebotomus socculus* | Finca Barranco, Almirante | Bocas del Toro | Buttresses in Cacao plantation | [63] |
| 23 | *Phlebotomus dasymerus* | Mojinga Swamp (CZ) | Panama | Mosquito light trap | [63] |
| 24 | *Phlebotomus hansoni* | Rio Corotu, Puerto Armuelles | Chiriqui | Buttressed roots | [63] |
| 25 | *Phlebotomus reburrus* | La Zumbadora, Cerro Azul | Panama | Shannon - light trap | [63] |
| 26 | *Phlebotomus pius* | Santa Clara, Volcan | Chiriqui | Buttresses in forest | [63] |
| 27 | *Phlebotomus botellus* | Volcan | Chiriqui | Mosquito light trap | [63] |
| 28 | *Phlebotomus rosabali n. sp.* | Puerto Armuelles | Chiriqui | Tree buttress | [59] |
| 29 | *Phlebotomus insolitus n. sp.* | Almirante | Bocas del Toro | Shannon - light trap | [59] |
| 30 | *Phlebotomus rubidulus* | Mojinga Swamp (CZ) | Panama | Mosquito light trap | [59] |
| 31 | *Lutzomyia tintinnabula n. sp.* | Altos del Quia | Darien | Mosquito light trap | [64] |
| 32 | *Phlebotomus viriosus* | Almirante | Bocas del Toro | Rock crevices | [61] |
| 33 | *Phlebotomus isovespertillionis* | Reared from eggs | Panama | Hollow tree with bats | [61] |
| 34 | *Phlebotomus carpenteri sp. nov.* | Chiva Chiva (CZ) | Panama | Mosquito light trap | [57] |
| 35 | *Phlebotomus runoides sp. nov.* | Almirante | Bocas del Toro | Between buttressed roots of forest trees | [57] |
| 36 | *Warileya rotundipennis sp. nov.* | Cerro Campana | Panama | Shannon - light trap in dense forest | [65] |

CZ = Canal Zone
